# Nature of Intestinal Secretions in Cholera

**Published:** 1847-12

**Authors:** 


*Nature of Intestinal Secretions in Cholera.*—The rice-water secretions in cholera have hitherto been looked upon as composed of the elements of the blood, albumen and serum being asserted to exist in the fluid portion, and fibrine in the solid parts. Chemical observation has now demonstrated to Prof. Andral the fallacy of this opinion. The fluid contained no albumen whatever, and the white grains floating in the liquid were not constituted by fibrine. Examined by the microscope, these particles presented the appearance of pus globules, but still they were not purulent. In the blood, albumen was found in its natural proportions. The learned professor concluded by stating as his opinion that the choleric matter consisted only of a modified mucous secretion.—*Med. Times, Aug.* 21, 1847.

